# Bone targeted nano-drug and nano-delivery

**DOI:** 10.1038/s41413-024-00356-2

**Published:** 2024-09-04

**Authors:** Yilun Wu, Bing Sun, Ying Tang, Aining Shen, Yanlin Lin, Xiaohui Zhao, Jingui Li, Michael J. Monteiro, Wenyi Gu

**Affiliations:** 1 College of Biotechnology and Pharmaceutical Engineering, Nanjing Tech University, Nanjing, China; 2 Australian Institute for Bioengineering and Nanotechnology, The University of Queensland, St. Lucia, QLD Australia; 3 Science and Technology Innovation Centre, Guangzhou University of Chinese Medicine, Guangzhou, China; 4 Shenzhen Bay Laboratory, Shenzhen, Guangdong China; 5 GMU-GIBH Joint School of Life Sciences, Guangzhou Medical University, Guangzhou, China; 6 School of Veterinary Medicine, Jiangsu Co-innovation Centre for Prevention and Control of Important Animal Infectious Diseases and Zoonoses, Yangzhou University, Yangzhou, China

**Keywords:** Bone cancer, Multihormonal system disorders

## Abstract

There are currently no targeted delivery systems to satisfactorily treat bone-related disorders. Many clinical drugs consisting of small organic molecules have a short circulation half-life and do not effectively reach the diseased tissue site. This coupled with repeatedly high dose usage that leads to severe side effects. With the advance in nanotechnology, drugs contained within a nano-delivery device or drugs aggregated into nanoparticles (nano-drugs) have shown promises in targeted drug delivery. The ability to design nanoparticles to target bone has attracted many researchers to develop new systems for treating bone related diseases and even repurposing current drug therapies. In this review, we shall summarise the latest progress in this area and present a perspective for future development in the field. We will focus on calcium-based nanoparticle systems that modulate calcium metabolism and consequently, the bone microenvironment to inhibit disease progression (including cancer). We shall also review the bone affinity drug family, bisphosphonates, as both a nano-drug and nano-delivery system for bone targeted therapy. The ability to target and release the drug in a controlled manner at the disease site represents a promising safe therapy to treat bone diseases in the future.

## Introduction

Tissue or organ targeted drug delivery holds the promise to increase the drug efficacy and reduce side effects. Unlike soft tissues, bone tissue possesses some specificity in cellular components, structure and distribution in the body, with unique features in disease development and drug treatment. Bone, for example, contains the major portion of calcium and phosphorus in the body and therefore, developing targeted drug delivery to bone will utilise these elements. Nano-delivery systems using calcium-based scaffolds are indeed very popular to repair bone defects and for targeted drug delivery. Bisphosphonates (BPs) are clinically used drugs for treating osteoporosis and bone cancers and frequently used as targeting motifs for bone targeted drug delivery due to their two phosphate groups and high affinity to bone. In addition, some delivery systems targeting biomarkers of bone diseases especially the bone cancers are also developed and reported, which can indirectly carry the drug to the disease site and diseased cells in bone tissue. All these delivery systems have boosted the development of bone targeted drug delivery and therapy. An ideal drug delivery system will require not only go to the disease tissue but deliver the therapeutic drug directly to the diseased cells.

In this review, we shall highlight and summarise the recent advances in these areas and propose new directions for future development of bone targeted nano-drug and nano-delivery systems. We will introduce bone related diseases and their treatments, with a focus on bone cancers including primary cancers and metastatic cancers. These cancers have no effective treatment methods and current treatments (including BP treatment) are far from satisfactory or with severe side effects.^1–3^ We will then review calcium homoeostasis of intracellular and extracellular environments and its metabolism in bone cells on regulating the osteogenesis or osteolysis followed by the use of different calcium compounds in regulating the bone microenvironment and their effects on disease (including cancer) development and progression. We will summarise nano-delivery systems and scaffolds using calcium-based materials.

In the third part of this review, we will review BP drugs as a drug delivery system. As a small molecule drug, the BP family has been explored as a popular bone targeting motif for many reported nano-delivery systems. The loading capacity of BP, however, is usually limited. Calcium-BP based nanodrugs and BP as a stabiliser for calcium-based nanoparticles (NPs) also show some exciting progress to prolong the short half-life in circulation of BP drugs. In the fourth section of this review, we shall review nano-delivery systems targeting the biomarkers of bone disease cells, including steroid hormones, specific markers to bone cancers and other bone diseases and special exosomes from bone cells, which also show bone targeted properties.

Finally, we conclude with a perspective of bone targeting nano-drug and nano-delivery systems as the dual targeting NPs on both bone and disease biomarkers or the multifunctional NPs with bone targeting and responsive components to bone diseases such as pH or temperature or released inflammatory cytokines.

## Introduction of bone diseases and their treatment

### Bone diseases and defects

Bones provide essential support and stability to the body, ensuring proper posture and movement. They form the framework that supports muscles, organs and tissues, allowing for functional movement and protection against external forces. Bone tissue undergoes constant remodelling, growth and repair in response to various factors such as mechanical stress, hormonal changes and metabolic demands. This dynamic nature allows bones to adapt to the body’s changing needs, whether it’s during growth and development, physical activity, or in response to injury or disease. This process involves the removal of old or damaged tissue and the formation of new bone tissue. It helps to repair micro-damaged areas, adapting the bone structure to changing loads and maintain minerals, especially calcium, homoeostasis.^4^

Bone diseases encompass a wide range of medical conditions that affect the skeletal system, compromising bone structure or defects, strength and overall health. The most common bone diseases include Osteoporosis, Osteoarthritis (OA), Rheumatoid Arthritis (RA), Paget’s Disease of Bone, Osteogenesis Imperfecta, Osteonecrosis, Fibrous Dysplasia, Rickets, Osteomyelitis and Multiple Myeloma.^5^ Treatments may involve medication, physical therapy, lifestyle modifications and, in some cases, surgical intervention. Bone metastatic cancers represent another large category of bone diseases, including osteosarcoma, which currently have no effective and specific treatment. Bone metastatic cancers occur when cancer cells from a primary tumour spread from other regions of the body to the bone. Such metastases can cause various complications, including pain, fractures and bone weakening. Though there are some treatment options, most treatments such as chemotherapy and radiotherapy are far from satisfactory with limited effectiveness and safety.

### Current treatment for bone disease and summary of bone targeted nano-delivery

Bone related diseases (including defects) cause major mobility hindrance and mortality in humans with no available effective treatments for most of them, especially for bone and bone metastatic cancers. There is a need to develop new drugs and/or bone-targeted drug delivery systems for more safe and efficient treatments.^2^ Osteoporosis is a very common bone disease and more often seen in menopaused women. It is generally managed with medications, including BPs, hormones, RANKL pathway inhibitors and others, that slow bone loss and reduce the risk of bone fracture.^6,7^ Although another bone anabolic hormone therapeutic, Forteo®, has the FDA approval, it is expensive requiring frequent doses that diminish the efficacy of the drug over time and increase side effects that include hypercalcemia, orthostatic hypertension and the risk of osteosarcoma.^8,9^ Prolia® is another FDA-approved monoclonal antibody that inhibits the RANKL pathway and prevents the proliferation and survival of osteoclasts and reduces bone resorption.^10^ Compared to BPs, Prolia® treatment may offer a cost-effective treatment for higher-risk and older adults with osteoporosis;^11^ however, it could lead to side effects of cellulitis, osteonecrosis of jaw and subtrochanteric fractures.^10^ Other osteoporosis therapies, such as oestrogen replacement therapy (ERT) and selective oestrogen receptor modulators (SERMs), inhibit osteoporosis by regulating osteoclast apoptosis but increase the risk of endometrial cancer, stroke and thromboembolism.^7,12,13^

For OA and RA, the pain management becomes essential using prescription pain relievers and using antirheumatic drugs to slow down the progression of rheumatoid arthritis and reduce inflammation.^14^ Biologic agents that target specific immune system processes can be effective in managing symptoms are also recommended.^15,16^ In these cases, a disease site specific drug delivery or localised drug delivery may be more efficient. For other bone related disease like Paget’s Disease of Bone, BPs are often used to help control bone turnover or in severe cases surgery can be applied to rectify deformities or fractures.^17^ For osteonecrosis, nonsteroidal anti-inflammatory drugs can help manage pain and inflammation.^18^ For osteomyelitis, intravenous or oral antibiotics are usually used to treat the infection.^19^ For multiple myeloma, chemotherapy or radiotherapy can be used like other bone related cancers. Meanwhile, the reduction of extracellular glutamate could significantly attenuate cancer-induced bone pain in mice model.^20,21^

Treatments for bone metastases aim to alleviate symptoms, prevent further bone damage and improve the patient’s quality of life. These treatments include pain management, radiation therapy, medications like BPs and denosumab to strengthen bones, surgery to stabilise weakened bones, systemic therapies and palliative care. According to statistics, the skeletal system is identified as the third most prevalent location for cancer metastasis, trailing only the lungs and liver^22,23^ with over 80% of the pain attributed to bone pain caused by metastatic cancer.^24^ The mechanism underlying bone cancer pain is notably intricate, encompassing various facets. These interactions involve various connections among tumour cells, bone cells, activated inflammatory cells and neurons innervated by bone nerves.^25^

The current primary approaches for pain management encompass non-opioid analgesics and opioid-based analgesics, bone-targeted interventions (NGF inhibitors; osteoclast inhibitors), as well as adjuvant therapies (corticosteroids, antiepileptic drugs). However, over 50% of individuals suffering from advanced or metastatic cancers are unable to attain satisfactory pain relief from these available cancer pain management strategies.^26^ In addition, most cancers developed to metastatic stage will have a poor prognosis and metastasis is a major cause of cancer related death.^27^ Therefore, development of new and effective treatment to bone metastatic cancers is an urgently clinical need.

To develop effective treatment and increase current drug efficacy in treating bone diseases and reduce side effects, bone targeted nano-drug and nano-delivery are promising. They may improve the treatment of various bone-related diseases and conditions, offering more effective and patient-specific therapeutic options. There are exciting advances in this field with various NPs delivering drugs (therapies) to different bone diseases (Table 1). It is expected that the advances in drug delivery systems and personalised medicine will revolutionise the management of bone-related diseases and health issues. These delivery systems will be introduced in detail in the following sections.

**Table 1:** Summary of nano-delivery systems for bone diseases

Bone diseases	NP used	Drug delivered	Drug efficiency	Ref
Bone Metastatic Cancer	Au_MSNs	ZOL	Increased	^197^
	ALN_PAMAM	DTX	Increased	^250^
	PLGA_ALN/FA_TPGS	PTX	Increased	^257^
	PLGA-PEG-ZOL	DTX	Increased	^199^
	MSNs_ZOL	DOX	Increased	^258^
	MSNs_ZOL	PL	Increased	^259^
	Ag2S_ALN	DOX	Increased	^260^
	PDA_ALN	SN38	Increased	^261^
	RGD	Bortezomib	Increased	^262^
Osteosarcoma	PEG	DOX	Increased	^263^
	Cu_MNSs	Cu, Si	Cell model only	^264^
	PLGA–nHAP	ZOL	Model	^265^
	iron oxide	Riluzole	Increased	^266^
Osteoporosis	MSNs_EDTA	17β-estradiol (E2)	Increased	^267^
	Nanostructure lipid carriers (NLCs)	RLX_VD	Increased	^268^
	Lipid nanoparticle	Simvastatin	Increased	^269^
	iPSC_Exosomes	SiRNA-Shn3	Increased	^270^
	nHAP co-doped with iron oxide	MiR-21/124	Increased	^271^
	nHAP	SCT	Increased	^272^
	ALN_mPEG_PLGA	Astragaloside	Increased	^273^
	PLGA loaded with secretome	CXCR4	Model	^274^
Bone regeneration	CS_TPP_nSiO2	NA	Model	^275^
	Intrafibrillar silicified collagen scaffold (SCS)	NA	Model	^276^
	Gd-MCS/CS	NA	Model	^277^
	CuS-PEG-PCL scaffold	Dexp	Model	^278^
	Lipid nanoparticle	SiRNA_GNAS	Model	^279^
Bone healing	ACP	ALN	Model	^280^
Tissue engineering	PLGA–nHAP	Plasmid DNA	Model	^281^
Osteoarticular tuberculosis	TC-PLGA-PEG	Rifapentine	Increased	^282^
Osteomyelitis	PLA_CS	Etoricoxib	Model	^283^
	Silver-copper-boron	Cadherin-11 antibody	Model	^284^
	mPEG-PLGA	Teicoplanin	Model	^285^

*ACP* Amorphous calcium phosphate, *ALN* Alendronate, *Au* Gold nanorods, *CS* Chitosan, *CuS-PEG-PCL scaffold* CuS nanoparticle-PEG soft hydrogel-coated 3D hard polycaprolactone scaffolds, *Dexp* Dexamethasone sodium phosphate, *DOX* Doxorubicin, *EDTA* Ethylenediaminetetraacetic acid, *FA* Folic acid, Gd Gadolinium, *nHAP* nano-hydroxyapatite, *MSNs* Mesoporous silica nanoparticles, *PAMAM* Polyamidoamine, *PDA* Polydopamine, *PL* Plumbagin, *PTX* Paclitaxel, *RGD* Arginylglycylaspartic acid, *RLX* Raloxifene hydrochloride, *SCT* salmon calcitonin, *SN38* 7-ethyl-10-hydroxycamptothecin, *TC* Tetracycline, *TPGS* D-α-tocopheryl polyethylene glycol succinate, *TPP* tripolyphosphate, *ZOL* Zoledronic acid

## Bone primary and metastatic cancers and current treatment

### Definitions and epidemiology

Compared to other bone diseases, bone related cancers have fewer treatment options and thus were emphasised in this review for bone targeted drug nano-delivery. Primary bone neoplasms are rare and account for only 0.2% of all human neoplasms.^28^ Bone tumours have a broad spectrum of morphology and biological behaviour. The fifth edition of the WHO classification of tumours of soft tissue and bone was published in April 2020^29^ and followed the fourth edition by the same organisation, describing the following lineage groups: (1) chondrogenic tumours, (2) osteogenic tumours, (3) fibrogenic tumours, (4) vascular tumours of bone, (5) osteoclastic giant cell-rich tumours, (6) notochordal tumours, (7) other mesenchymal tumours of bone and (8) hematopoietic neoplasms of bone. Besides bone primary cancers, bone is one of the most common metastatic sites for cancers, especially in the lung, breast and prostate.^30^ The level of bone metastasis is strongly linked with shorter survival rates.^31^ Bone is one of the most common sites of metastasis from advanced solid cancers and bone metastases occur in 65%–80% of patients with advanced prostate or breast cancer, 40%–50% patients with lung cancer and in <10% of those with gastrointestinal cancer.^32^

A study, including 1 849 patients with bone metastases arising from seven major types of cancers who were enroled from the Korean National Sample Cohort database, found that 8.6% of the newly diagnosed cancer patients had bone metastases.^33^ The overall occurrence of bone metastasis from solid cancers was similar to that reported from other countries. In 2004, Schulman et al. reported that 5.3% of cancer patients in the United States (US) had metastatic bone disease.^34^ A Danish population-based cohort study of registered patients diagnosed with cancer between 1994 and 2010 showed that 7.5% of patients with prostate, breast and lung cancer, which were the three most common primary cancers out of 10 cancer types, had metastatic bone disease.^30^ Similarly, a retrospective cohort study conducted in Thailand from 2006 to 2015 demonstrated that 7.7% of the patients diagnosed with the 10 most common cancer types had bone metastases.^35^ The incidence of bone metastasis according to the cancer type differed between countries. However, most of the previous studies showed that the top three leading cancers associated with the development of bone metastasis were prostate, breast and lung cancers.^36^ Less than 5% of stomach and colorectal cancer patients developed bone metastasis, which is consistent with the historical data (3%–5%).^37^

Previous studies have found that the mean time from the primary cancer diagnosis to the development of bone metastasis is 18.9 months and the incidence of simultaneous diagnosis of the primary tumour and bone metastasis was 25.4%.^38^ This is consistent with the findings from a US study, which reported that the incidence of bone metastasis in patients with solid cancers typically increases during the first 2 years.^38^ The current treatment for bone cancers is summarised in Table 2.

**Table 2:** Treatments to bone cancers

Classification		Treatment
RIMARY BONE TUMOUR	Benign bone tumour	Tumour removal, bone restoration
	Malignant bone tumour	Limb-sparing surgery, amputation, chemotherapy, radiotherapy
	Borderline bone tumour	High malignancy risk-consider lymph node dissection or amputation Local therapy or radio/chemo; Traditional Chinese Medicine (TCM)
	Bone marrow and hematopoietic system tumours	Pain relief, infection treatment, blood transfusions, electrolyte balance correction; Chemotherapy; Radiotherapy; Surgery
	Neoplasmoid lesion	Surgery if discomfort from suspected malignancy
SECONDARY BONE TUMOUR	Metastatic tumour	Whole body treatment options: Chemotherapy, targeted therapy, endocrine therapy, immunotherapy and TCM. Local treatments: surgery, radiotherapy, radionuclide therapy and interventional therapy; Drug therapy: bisphosphonates, analgesics, etc.

There is currently no effective treatment for cancer metastasis, the major cause of cancer related death.^27^ Development of new treatments for bone metastatic cancers with bone targeted drug delivery is now an important area of development.^2,39^ Since bone contains large amounts of calcium, its role in bone disease development (including cancers) and as a bone targeted delivery system is described first.

## Calcium metabolism in bone diseases and calcium-based nano-delivery

With the rapid development of nanotechnologies, versatile nanomaterials have emerged for disease treatment and diagnosis. Nanomaterials enable the payload of numerous bioactive molecules to achieve enhanced treatment efficacy, by encapsulating hydrophobicity drug, protecting them against enzymatic degradation, facilitating the phagocytosis of specific cells and enhancing the accumulation at the lesion site. The intrinsic physical and chemical properties of nanomaterials exert additional modulation potency to reshape the local physiological environment. Inorganic nanomaterials regulate bioprocesses, endowing element-enabled photothermal effect, reactive oxygen species (ROS) generation, metabolic interferences and extended applications in diagnosis. Taking advantage of the basic and most abundant element in bone tissue, calcium-based nanomaterials hold may be harnessed for bone disease treatment and anti-bone cancer agent delivery.^40^

### Calcium signalling in bone disease

#### Calcium homoeostasis

**Fig. 1:**
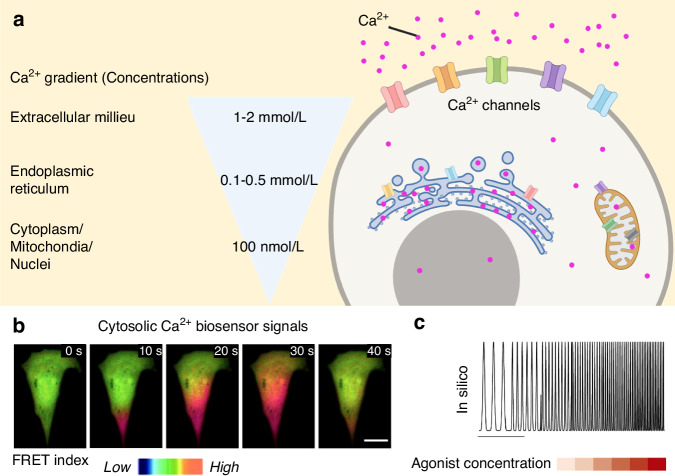
Calcium homoeostasis and oscillation. **a** Calcium gradient under resting conditions. **b** Intracellular calcium signals detected by a cytosolic FRET-based calcium sensor suggesting the calcium oscillation occurs in hMSC. Red and blue colours represent the high and low FRET ratios, respectively. Images were captured in 10 s interval, scale bar, 20 μm.^286^ **c** A computer-generated Ca^2+^ oscillatory wave frequency modulated by agonist concentration.^50^ (**a** Created with BioRender.com. Reprinted with copyright permissions)

Under resting conditions, the distribution of calcium ions (Ca^2+^) shows a concentration gradient between cell and its surroundings. As shown in Fig. 1a, the extracellular milieu maintains a high Ca^2+^ concentration about 1–2 mmol/L, whereas the intracellular Ca^2+^ is around 100 nmol/L.^41–43^ The Endoplasmic reticulum (ER) is responsible for the dynamic storage of intracellular Ca^2+^, resulting in a distinct high Ca^2+^ concentration compared to the surroundings. In addition, endo-lysosomes also contain high Ca^2+^ concentration, ranging from 0.4 to 0.6 mmol/L with high sensitivity to pH.^44,45^ Meanwhile, mitochondria are also regarded as calcium storage organelles, holding a Ca^2+^ storage capability up to 0.5 mmol/L with stimulus.^46,47^ Alleviating their intermembrane space and matrix, mitochondria absorb Ca^2+^ to generate energy, form modulation signals and regulate cell apoptosis.^48^

The Ca^2+^ gradient is controlled by a series of Ca^2+^ channels on the cell membranes and on the membrane of ER and mitochondria. As seen in Table 3, Ca^2+^ transfers into the cytoplasm, mitochondria and ER through voltage-gated Ca^2+^ channels (VGCs), transient receptor potential channels (TRPs), store-operated calcium channels (SOCs), ion-exchange pumps and other forms of channels. These channels enable the Ca^2+^ diffusion under stimulus to transduce signals and modulate cellular responses. The transportation of cytoplasmic Ca^2+^ between several channels initiates wave-like periodical signals in both excitable and non-excitable cells, or generally termed as ‘calcium oscillation’ (Fig. 1b).^49^ Bioinformation can be subsequentially encoded by the oscillatory wave’s parameters such as amplitude, frequency, period and duty cycle. Any external stimulations would be transcoded into calcium signals by proportional changing the parameters. As an example showed in Fig. 1c, the external stimulation of an agonist modulate the calcium oscillation in a dose-dependent manner in silico.^50^ Thereafter, the calcium oscillation will be sensed and decoded by effectors, including NFAT (nuclear factor of activated T-cells), NF-κB (nuclear factor kappa B), CaMKs (Ca^2+^/calmodulin-dependent protein kinases), PKC (protein kinase C), calpain and Ca^2+^-sensitive mitochondrial dehydrogenases.

**Table 3:** Major calcium channels, transporters, pumps and exchangers

Channels	Description
Ca_v_s	Voltage-gated calcium channels, transducing membrane potential changes into intracellular Ca^2+^ transients for intracellular signal transduction
TRPCs	Canonical transient receptor potential channels on cell membranes for calcium influx
TRPMs	Transient receptor potential melastatin on cell membranes for calcium influx
TRPVs	Transient receptor potential vanilloid on cell membranes for calcium influx
ORAI1/2	Calcium release-activated calcium channel protein 1/2, a Ca^2+^ selective ion channel on cell membrane that encoded by *ORAI1* gene, interact with STIM1 to activate store-operated Ca^2+^ entry (SOCE)
STIM1	Stromal interaction molecule 1 that locates on the ER membranes. Activated STIM1 oligomer translocate to bind activated ORAI1/2 to form store-operated calcium channel (SOC) complex for calcium influx
NCX	Sodium-calcium exchanger pumps, responsible for the exchange of cytosolic Ca^2+^ and Na^+^ at the extracellular milieu with a stoichiometry of 1:3. When expressed on the inner membrane of mitochondrial, it pumps Ca^2+^ out to the intermembrane spaces and pumps Na^+^ into the matrix.
MCU	Mitochondrial calcium uniporter, locating on the outer membrane of mitochondrion and assisting the uptake of Ca^2+^ from cytosol
IP_3_R	Inositol trisphosphate receptor that responsible for the Ca^2+^ release from ER and the oscillation signals
PMCA	Plasma membrane Ca^2+^ ATPase that belongs to the P-type primary ion transport ATPase, pumping out cytosolic Ca^2+^ by ATP hydrolysis at a stoichiometry of 1:1
SERCA	Sarcoplasmic/endoplasmic reticulum Ca^2+^ ATPase, allowing the transport of Ca^2+^ from cytosol into ER in an APT consuming manner.

#### Intracellular calcium and cellular processes

**Fig. 2:**
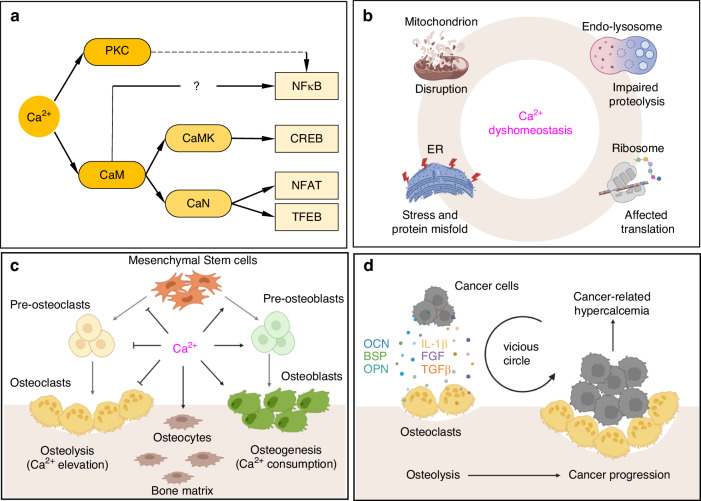
The effects of cellular calcium on bone diseases. **a** Intracellular calcium signalling in regulating key bone metabolic processes. Elevated intracellular calcium affects critical bone metabolic processes. **b** Intracellular calcium concentration interacts with various subcellular organelles including mitochondrion, endo-lysosome, endoplasmic reticulum (ER) and ribosome. **c** Extracellular calcium concentration modulates bone metabolism. **d** The vicious circle between tumour cells and the local bone-related cells in bone cancer progression interfere with bone matrix proteins and cytokines secretion and causes cancer-related hypercalcemia. Figure created with BioRender.com

Intracellular Ca^2+^ is involved in the regulation of gene expression as a second messenger. As shown in Fig. 2a, there is an elevation of intracellular Ca^2+^ activate calcium binding messenger proteins such as protein kinases C (PKC) and calmodulin (CaM). Indeed, the activation of PKC pathway is strongly associated with its upstream phospholipase C (PLC) activation, especially Wnt signalling controlled PLC activation in bone tissues. PLC catalyses the hydrolysis of phosphatidylinositol 4,5-bisphosphate (PIP_2_) into inositol trisphosphate (IP_3_) and diacylglycerol (DAG). The former is associated with Ca^2+^ release to cytoplasm from ER and the latter is associated PKC recruitment. PKC signalling is indispensable in both chondrogenesis and osteogenesis.^51^ Early activation of Ca^2+^/PKC was able to determine the bone formation from non-bone forming constructs.^52^ In a typical PKC signalling process, the interacting protein p62 is upregulated and forms a ternary complex with TRAF6 and Ca^2+^ activated PKC to orchestrate RANKL signalling and osteoclastogenesis.^53,54^ On the other hand, CaM activates calcineurin (CaN) and calmodullin-dependent kinase (CaMK) and subsequently regulate the downstream transcription factors including nuclear factor of activated T cells (NFAT), transcription factor EB (TFEB), cAMP-responsive element-binding protein (CREB), myocyte-specific enhancer factor 2 (MEF2) and nuclear factor kappa B (NF-κB). These CaM modulated signals are critical in bone formation. As previously reported, bone mass and quality dependent on osteoblast-lineage Ca^2+^/CAMKII.^55^ The interaction of CaM with Rab3D is required for osteoclast-mediated bone resporption.^56^ Taken together, calcium signalling is at the intersection of critical bone metabolic processes such as osteogenesis and osteolysis.

Intracellular Ca^2+^ concentration also shows complicate intersections with a variety of subcellular organelles. Thus, the imbalance of calcium impairs cellular processes through organelle dysfunctions (Fig. 2b). As the main Ca^2+^ depot, ER ensures intracellular Ca^2+^ homoeostasis by store-operated Ca^2+^ entry (SOCE) ubiquitously in excitable and non-excitable cells. The SOCE process depends on the coordinated activity of two families of proteins, STIM and ORAI, with details listed in Table 3. Exorbitant calcium perturbation is engaged in disrupted ER function. During the so-called ‘ER stress’, specific signalling is triggered as response strategies, including the unfolded protein response (UPR), the ER-overload response and the ER-associated degradation.^57–59^ As a results, a cell under ER stress will lead to accumulation of misfolded proteins, increase of protein degradation, reduction of client protein processing and ultimately cell apoptosis.^60^ In mitochondrion, Ca^2+^ entry into the matrix through the mitochondrial calcium uniporter to facilitate ATP production by altering the activity of matrix enzymes and the inner mitochondrial membrane potentials.^61,62^ Simultaneously, ROS is produced as by-products during this process. Excess Ca^2+^ is implicated with ROS induced mitochondrial disruption, resulting in imbalance of intracellular oxidative stress and finally cell apoptosis.^63,64^ Endo-lysosomes are a series of vesicle organelles participating the endocytic pathway, playing essential roles in protein transport, membrane trafficking, autophagy and exogenous cargo lysis. Recent studies attempt to link their functions with Ca^2+^ signalling. Endo-lysosomes employ H^+^/Ca^2+^ exchanger for calcium influx and proton efflux. So, the dyshomeostasis of calcium is suspected to be associated with pH control in endo-lysosomes for the functions of their lysozymes. Through ryanodine receptors (RyR), excess Ca^2+^ can be transferred from ER to endo-lysosomes to avoid ER stress. However, this process is observed to link with the reduction of proton pump vascular ATPase (vATPase), resulting in the deacidification and impaired proteolysis.^65,66^ Similarly, pH-dependent calcium channel RECS1 induces cell death through canonical mitochondrial apoptosis pathway by regulation of endo-lysosomal acidification and calcium content.^67^ In addition, calcium dysregulation also affects ribosomal function by triggering the dissociation of myosin-Va.^68^

#### Extracellular calcium and bone diseases

Bone metabolism, or bone remodelling, refers to the constant dynamic process of osteoblast-mediated osteogenesis and osteoclast-mediated osteolysis. Dysregulation of this process is associated with a wide range of bone diseases. The perturbation of extracellular Ca^2+^ is linked to the activities of bone metabolism, as mentioned in above section.

In general, extracellular free Ca^2+^ concentration in bone site at a steady state is maintained 1.1–1.3 nmol/L, which is similar to that of other tissues despite the fact that bone tissue is the major calcium storage site.^69^ Most of bone calcium is stored through mineralisation in the form of hydroxyapatite. During bone metabolism, activation of osteoclasts causes the dissolve of mineralised bone matrix to elevate extracellular Ca^2+^, whereas activation of osteoblasts triggers bone formation and Ca^2+^ consumption. In turn, extracellular Ca^2+^ promotes osteogenesis process by activating osteoblasts and inhibiting osteoclasts as a feedback loop.^53,70^ Moreover, high extracellular calcium regulates the formation of osteoblasts and osteoclasts by modulation of the maturation and differentiation of mesenchymal stem cells (MSCs) and precursor cells.^71,72^ The effects of Ca^2+^ on bone metabolism is schematically summarised in Fig. 2c.

During primary oncogenesis or metastasis progression at the bone site, tumour cells hijack osteoclasts to reshape the bone microenvironment.^73^ As in Fig. 2d, a ‘vicious cycle’ of crosstalk between tumour cells and the local bone-related cells is established when bone tumour progression, especially in the scenario that breast cancer or prostate cancer metastatic to the bone site. In this process, tumour cells initially perform osteoblast mimicry to adjust themselves from a hostile tissue by producing bone matrix proteins (such as OCN, BSP and OPN).^74^ Simultaneously, tumour cells manipulate direct secretion of cytokines (such as IL-1β, FGF, TGFβ and TNFα) and indirect induction of osteoblast-produced RANKL to orchestrate the formation and activation of osteoclasts.^75^ Finally, DTC invade the bone site by alteration of bone metabolism. Because of the bone resorption, hypercalcemia is often observed in patients with bone cancer or bone metastasis and is associated with a poor prognosis.^76,77^

### Calcium-based nanomaterials in bone disease treatment

Calcium-based nanomaterials are widely applied in the treatment of bone diseases. Indeed, the bone tissue matrix is abundant in several calcium-binding proteins, resulting in the high affinity to calcium-based nanomaterials and the loaded therapeutic agents. These nanomaterials also serve as calcium supplements to benefit the osteogenesis process. Notably, recent studies in bone cancer or bone metastasis unveiled the unique functions of calcium in cancer theragnostic. Here we will review some of the pioneer works utilising calcium-based nanomaterials for bone disease treatment.

#### Chemistry composition

In general, the commonly used calcium nanomaterials include calcium phosphate (CaP), calcium carbonate (CaC), calcium fluoride (CaF) and bioglasses (BGs). The differences in chemistry composition may bring some unique characters based on the anion group, such as the gas-generation ability of CaC, autofluorescence phenomenon of CaF, oxidant reduction regulation ability of CaH_2_ and CaO_2_. Chemistry composition of calcium nanomaterials also endows with distinct mechanical and physical properties such as stoichiometry, stiffness and even solubility. These properties alter between different crystal structures. As reported, CaP-based materials (around 5 for apatite) show higher Moh’s mineral hardness than CaC-based materials (around 2.5-3 for calcite), with great variations between different crystals.^78,79^ The hardness similarity of CaP to bone tissue (about 5 in Moh’s hardness) endows their fitness as a substitution scaffold. Hydroxyapatite (HA), with a Ca/P ratio of 1.67, shares the closest composition to natural bone mineral,^80^ followed by tricalcium phosphate (TCP) and biphasic calcium phosphate (BCP), another two formats that commonly applied in bone biology. Some review articles suggested the osteoinductive potential follows a trend of BCP > TCP > HA in most cases, while they also claim the existence of some conflicting findings.^81,82^ Indeed, compared to a definite single composition, calcium-based mixture compositions, such as ceramics and bioglass (Ca-BG) and some cements are more ubiquitous in the construction of bone scaffolds. The basic calcium component of common cements, ceramics and Ca-BGs ensures these materials with appropriate mechanical characteristics in bone substitution application and bioactive interfaces to interact with osteocytes. Numerous investigations have suggested their roles in osteogenesis induction, osteoclastogenesis restriction and some influence on angiogenesis to facilitate the new bone formation, as summarised in some enlightening review articles.^83,84^ The unique applications of CaH_2_ and CaO_2_ on bone related cancer will be discussed later in the section “Scaffold-free calcium nanomaterials for osteoporosis and bone defects”.

#### Nanoscale geomorphology of calcium-scaffolds

In the treatment of diseases caused by bone mass loss, the materials are required to possess osteogenic capacity for bone regeneration and mechanical strength for bone support. As known, bone scaffolds are widely used as an implantation to treat bone defects. Meanwhile, in some scenario such as osteoporosis, such scaffolds are also required to prevent or protect against bone fraction. Calcium-based materials, with a similar chemical composition of the bone tissue, show good osteoconduction, osteoinduction and osteogenesis abilities,^85^ making them ideal materials as the main composition of bone scaffolds.

**Fig. 3:**
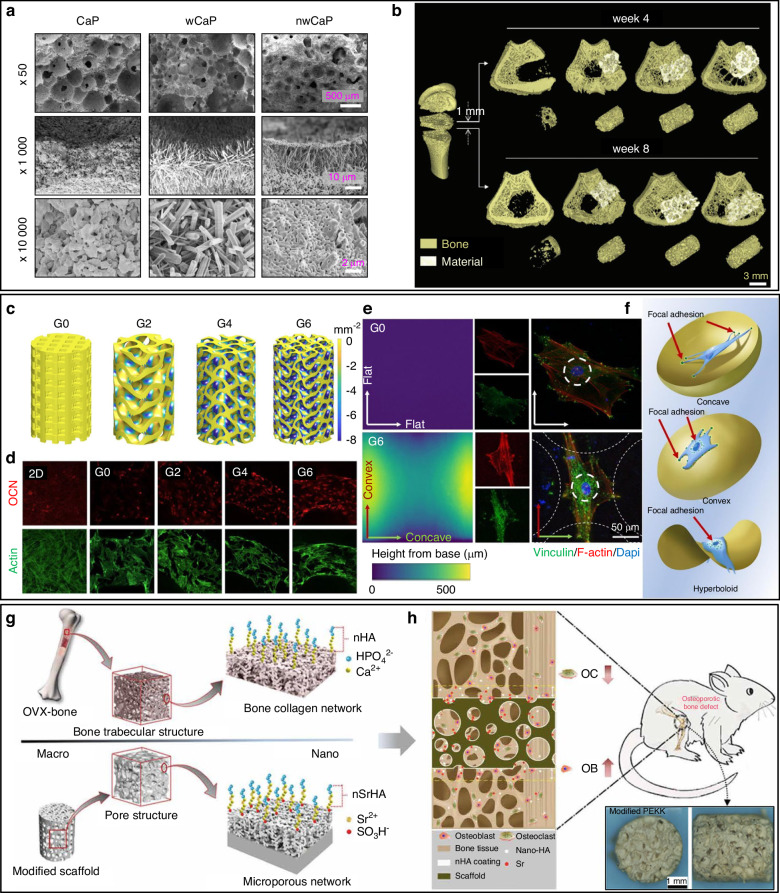
Calcium scaffolds with nano- and macro-structures for bone repair. **a** The SEM analysis of calcium phosphate bioceramics (CaP), CaP with a micro-whiskers structure (wCaP) and micro- /nano- CaP (nmCaP); **b** The three-dimensionally reconstructed micro-CT images of femoral bone defect model showed the implanted materials (white) and newly formed bone (yellow). More bone was formed in nwCaP and wCaP.^90^ **c**–**f** The calcium scaffold with different material surface curvature (G0, G2, G4 and G6) modulated osteoclacin (OCN) expression, more vinculin around nucleus and cell contractility.^97^ **g**, **h** The SO_3_H^-^ coated polyetheretherketon (PEEK) affected osteogenesis and osteoclastogenesis of osteoporotic bone defect.^101^ (Reprinted with copyright permissions)

The construction of calcium-scaffolds with unique nanoscale geomorphology is critical, as the nanoscale structures help to exert better cell functionality than those with micro or macroscale structures.^85,86^ These micro or macroscale structures can be fabricated with the aids such as emulsion restriction, hydrothermal conditions, precursor/template guidance and mechanical activation.^87–89^ In general, these approaches lead to unordered structures on the interface of calcium materials. For example, ref. ^90^ modified porous calcium phosphate (CaP) ceramics with micro-whiskers structure (wCaP) and micro/nano-structured CaP (nwCaP) by nanoparticle deposition (Fig. 3a). The micro-CT reconstruction data suggested nwCaP with this unique micro/nanostructure can reduce the fracture occurrence and induce substituted bone formation in a rat model with osteoporotic bone defects (Fig. 3b). Apart from unordered nanoscale structures, calcium materials with ordered periodic nanopatterns offer opportunities to induce the cell differentiation by control the orientation of its growth. The nanopattern is also a mimic to the natural bone microenvironment. As reported, bone formation starts from the apatite crystals nucleate in the 40-nm-long gap zone of the periodic 67 nm cross striated pattern of collagen fibril.^91^ In addition, the periodic patterns of nanomaterials may further be used to decode bone formation bioinformatics by utilising their structural colous.^92–94^ Plant tissues with natural porous structures may further inspire the scaffold design for bone regeneration.^95,96^

Alteration of surface curvature of materials with similar/same microcosmic structure/patterns is recognised to have impacts on cell behaviours. Based on this notion, ref. ^97^ fabricated three dimensional scaffolds with triphy periodic minimal surfaces (TPMSs) with hyperboloidal structure on ever surface point with varying Gaussian curvatures using calcium phosphate. Gyroid TPMS scaffolds with different average Gaussian curvatures of 0, −2, −4 and −6 mm^−2^ (denoted as G0, G2, G4 and G6) were designed and fabricated (Fig. 3c). All scaffolds with Gaussian curvatures exhibited enhanced osteogenic potentials than the G0 and 2D scaffolds in human mesenchymal stem cells (hMSCs), showing increased expression of osteoclacin (OCN) as well as other markers (Fig. 3d). The immunofluorescent visualisation of the stress fibres (SFs) and the focal adhesion (FA) complex demonstrated the hMSCs presented elongated orientation of SF in the convex direction, decreased cell area and increased aspect ratio, suggesting the cell contractility modulation (Fig. 3e), as schematically illustrated in the Fig. 3f. Taking advantage of this TPMS scaffolds, the new bone formation and angiogenesis effect were augmented in the animal model.

Due to the diversity of clinical demands, not all the scaffolds applied in bone tissue engineering are fabricated by calcium-based materials. Nevertheless, calcium-based materials can benefit by providing a biocompatible coating material. Thermoplastic polymers, such as polyetheretherketon (PEEK), polycaprolactone (PCL), poly-lactic-glycolic acid (PLGA) and blending polymers, are feasible in a variety of sophisticated scaffold fabrication methods like 3D printing and electrostatic spinning.^98–100^ However, their clinical application in bone diseases hampered by their poor osteointegration with host bones. To overcome this drawback, the surface of PEEK scaffolds was modified by HA microsphere, sulfonated to introduce the SO_3_H^-^ groups, grafting with Sr^2+^ and finally adopted hydrothermal treatment for size and crystallinity control (Fig. 3g).^101^ The obtained scaffolds with HA and Sr^2+^ coating promoted new bone formation and reduced adjacent bone loss by the synergy of enhancing osteogenesis and inhibiting osteoclastogenesis.

#### Scaffold-free calcium nanomaterials for osteoporosis and bone defects

Apart from scaffolds, calcium nanoparticles or nanosheets can be directly employed in the osteoporosis and bone defect treatment. Despite the aqueous solution of these calcium nanomaterials cannot provide technical support to the bone tissue, the nano formulation reserves high drug payload capacity compared to the solid ceramics and bioglass applied in bone scaffolds. Moreover, nanoparticles and nanosheets are easily metabolised in vivo, featured with quicker local dissolution kinetics in situ and quicker systematic clearance from the bloodstream. In addition, most of the calcium nanomaterials are alkaline precipitates. Their intrinsic antacid properties can be utilised to reshape the osteoporotic microenvironment by neutralising the accumulated acid from over-activated osteoclasts. Taken together, the calcium nanomaterials serve as nano-drugs to inhibit pathological osteolysis and further induce osteogenesis.

**Fig. 4:**
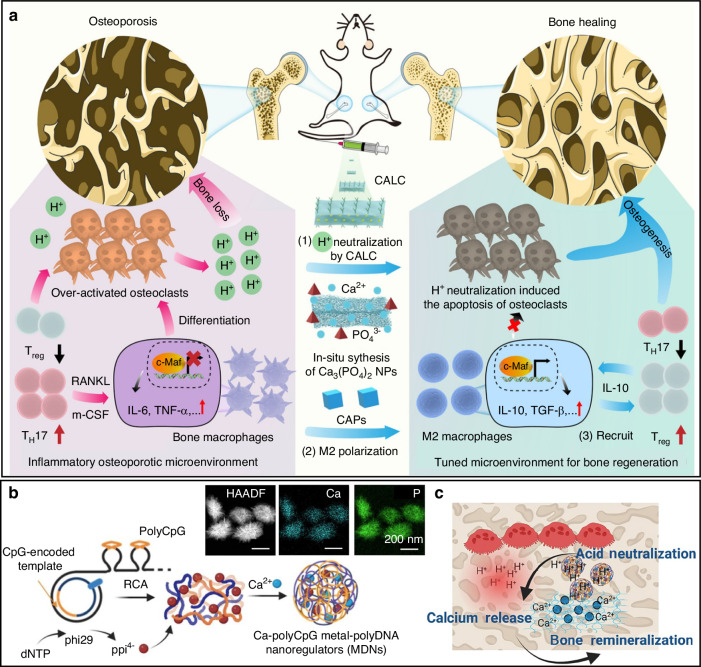
Calcium based nanoparticles for ameliorating osteoporosis. **a** The calcium-aluminium layered double hydroxides (CALC) modulate osteoporosis through neutralising pH and regulating immune environment including macrophage repolarization and Treg recruitment.^102^ **b**, **c** The metal-poly DNA nanoparticles consisting calcium exert acidic neutralisation, calcium release and bone remineralization against osteoporosis.^107^ (Reprinted with copyright permissions)

Layered double hydroxide (LDH) are characterised by their sheet stacks. Typically, these nanosheets consist with a trivalent metal ion, a divalent metal ion and a replaceable anion that can be used for drug payload by ion exchange. To effectively reverse osteoporosis, a specially designed nanocatalytic medicine was fabricated by loading bone targeting group calcein to a calcium-aluminium LDH (CALC).^102^ As shown in Fig. 4a, CALC neutralised acidic microenvironment to suppress osteoclast activities at the lesion site and release Ca^2+^ for the synthesis of CaP nanoparticles in situ with endogenous phosphate cations. Simultaneously, CaPs modulated the M2-like macrophage polarisation via c-Maf signalling and secretion of inflammatory cytokines such as IL-10 and TGFβ. Finally, T_reg_ cells were recruited to inhibit the activation of T_H_17. With the synergy of bone and immune microenvironment regulation, calcium based CALC nanomedicine exhibited impressive therapeutic efficacy to aged mice with osteoporosis. Taking advantages of the Ca^2+^ high affinity to oligonucleotides, functional DNA or RNA can be introduced with calcium nanoparticles in bone research. Taking the advantages of the porous structure and metal chelating abilities, meta-organic framework (MOF) is widely investigated to extend their applications in bone diseases.^103–106^ As in Fig. 4b, a therapeutic MOF structure consisting calcium ion and poly CpG DNA was prepared and customised for osteoporosis treatment.^107^ To overcome the poor stability and affinity in the biological environment, an ultralong single-stranded DNA with polyvalent functional DNA sequences was applied to coordinate with the calcium ion to form multifunctional metal-poly DNA nanoparticles (MDNs). After injection, the acidic microenvironment was neutralised by MDNs, triggering the Ca^2+^ release and sequential mineralization in porous bone tissue (Fig. 4c). Synergistically, polyCpG induces the local elevation of IL-12 to inhibit osteoclast differentiation and effector proteins. In another work, miRNA (miR-210) and a treatment agent simvastatin were loaded to the CaP nanoparticles for facilitation of angiogenesis and osteogenesis, respectively.^108^ This design provides a novel strategy for bone repair based on the powerful payload capacity of CaP nanoparticles.

#### Therapeutic and diagnostic effects of calcium nanomaterials in bone cancers

**Fig. 5:**
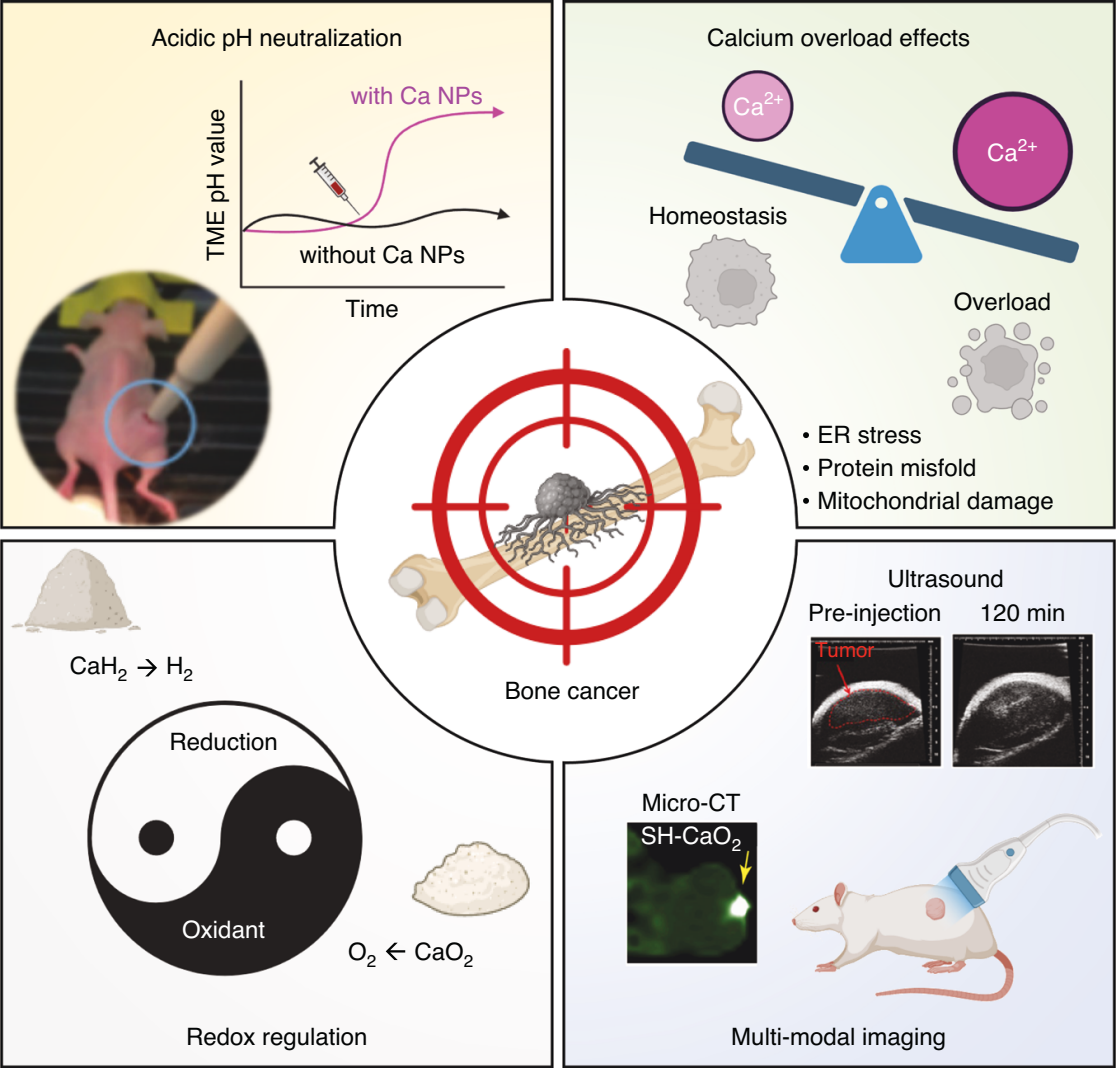
Calcium-based materials for bone cancer therapy and diagnosis. The calcium-based nanomaterials regulate tumour microenvironment and facilitate tumour diagnosis through neutralising acidic pH in tumour microenvironment, causing calcium overload in cancer cells, regulating redox in tumour conditions and elevating diagnostic efficacy. Mouse photo,^93^ ultrasound^145^ and micro-CT images^122^ were modified and reprinted with copyright permissions. Figure created with BioRender.com

Malignant oncogenesis that originated from the bone tissue form primary bone cancer, including osteosarcoma, chondrosarcoma, Ewing sarcoma and chordoma. Besides, bone is regarded as vulnerable to be invaded by tumour from other organs, especially those metastatic from breast, lung and prostate. As discussed in “Extracellular calcium and bone diseases”, bone microenvironment is reshaped to tolerate tumour growth. Taking advantage of their high affinity to the bone tissue, calcium-based nanomaterials can serve as a delivery platform for efficient anticancer drug delivery in bone cancer treatment, Moreover, these materials fascinate researcher’s attentions via their intrinsic effects on therapeutic and diagnosis, as summarised in Fig. 5.

The tumour microenvironment (TME) is featured with a mild acidity due to the accumulation of metabolic products such as pyruvate and lactate.^109^ As reported, the average extracellular pH of sarcoma is about 6.7, whereas that of skeletal muscle is 7.35.^110^ Neutralisation of tumour extracellular pH lead to the metabolism alteration in the TME, including inhibition of tumour glycolysis,^111^ prevention of metastasis,^112^ induction of M1-like macrophage polarisation,^113^ unblockage of T cell immunity,^114,115^ reprogramme of epithelial-to mesenchymal transition,^116^ and suppression of cancer associated fibroblast phenotypes.^117^ The alkaline calcium-based nanomaterials can regulate the pH after injection. For example, in nude mice bearing HT1080 tumour, Intravenous injection of CaCO_3_ nanoparticles can increase tumour pH from 7.23 to 7.4 and repeat administration of these drug-free CaCO_3_ nanoparticles showed inhibition to the tumour growth in vivo.^118^ The effective concentration to induce pH change in the TME differs between nanoparticles with different chemical compositions due to their distinct dissolution profiles with pH changes. Physical properties like particle interfaces also count for the pH modulation ability.

Due to the effective tumour accumulation, calcium nanomaterials elevate Ca^2+^ concentrations in tumour TME and eventually aggrandise the intracellular Ca^2+^. In this case, organelles response for calcium storage and regulation will be exposed to a danger state called calcium overload, referring to the long-term calcium dyshomeostasis. Under this status, cancer cell suffers from ER stress, irregular protein fold, imbalanced intracellular redox and insufficient ATP production, abnormal cell signalling and finally lead to the apoptosis. Calcium overload can be observed in cells treated with various calcium nanoparticles. Li et al. ^119^ fabricated a nanomedicine using CaCO_3_ incorporation with a biosafety flavone kaempferol-3-O-rutinoside (KAE). In this design, KAE exerted anticancer ability and facilitate calcium influx, while CaCO_3_ serves as an ideal calcium ion supplier to augment the calcium overload in cancer cells. To precisely induce intracellular calcium overload, Dong et al. ^120^ synthesised a pH-dissociable hollow coordination CaCO_3_ nanoparticles. The so-called BSP-TCPP/Fe@CaCO_3_-PEG nanoparticles integrated a sonosensitizer (TCPP), a metallic centre (Fe^2+^), a glutathione (GSH) biosynthesis inhibitor (BSO) and a calcium supplier (CaCO_3_). Under ultrasound exposure, such nanoparticles showed effective cell killing ability by inducing calcium overload and oxidative stresses. To initiate calcium overload-related cell death, the Ca^2+^.concentration should be at least 20 μg/mL in most reports.^121,122^

Specific calcium materials enable the intracellular redox regulation, including calcium peroxide (CaO_2_), calcium hydride (CaH_2_) and calcium fluoride (CaF_2_). CaO_2_ enables the release of Ca^2+^ and generation of H_2_O_2_ under acidic pH, exerting as an oxidant in the tumour conditions. The hydrolysis of CaO_2_ leads to O_2_ generation, alleviating the hypoxia in the TME and suppressing tumour-associated glycolysis. Therefore, the CaO_2_ exerted a combination of the following anticancer mechanism: calcium overload, ROS generation and oxygen generation. Moreover, Fenton-type metal ions can be incorporated in the CaO_2_-based nanoparticles to enhance tumour therapies.^123–125^ Recent studies have shown some paradigms on how to engage Cu^2+^, Fe^3+^ and Mn^2+^ to catalyse the H_2_O_2_ produced by CaO_2_ for the generation of cytotoxic hydroxyl radicals.^126–129^ CaH_2_ is generally used as a reducing agent or a drying agent. The grey powder of CaH_2_ is easily to hydrolyse, with quick and vigorous hydrogen gas generation. H_2_ acts as a therapeutic antioxidant by selectively reducing the oxygen radicals, thus benefiting the treatment of oxidative stress- and inflammation-related diseases in the clinic, including cancer.^130,131^ Therefore, the application of CaH_2_ provide a promising treatment concept based on the functions of H_2_ and Ca^2+^ in cancer therapies. Recently, Gong et al. ^132^ reported a facile and straightforward method to synthesise nanoscale CaH_2_ particles by liquid-phase exfoliation technology. The obtained CaH_2_ was dispersed in polyethylene glycol (PEG) with a low average molecular weight of 200 for storage and intravenous administration, thus protecting against water contact and hydrolysis. Compared to the in situ photocatalysis methods for H_2_ generation,^133–135^ the application of CaH_2_-based nanomaterials overcomes the depth limitation by light penetration. Future investigations may focus more on developing tumour-specific detachment of the water-proof coating on the CaH_2_ particles for precision and efficient H_2_ generation. Alternatively, CaSi_2_ also generate H_2_ when hydrolysis. By fabricating CaSi_2_-containing polyhydroxyalkanoate into mesoporous BGs using electrospray, the scaffolds can achieve sustained and local H_2_ release after implantation in mice to improve bone repair.^136^ CaF_2_ is a ubiquitous natural mineral with autofluorescence under UV excitation, from which arising the name, fluorite. Indeed, CaF_2_ holds potential to catalyse and delete peroxides and breakdown the intracellular redox balance. This peroxidase (POD)-mimicking activity can be amplified by ultrasound, accompanying with the release of exogenous Ca^2+^. to achieve calcium overload effect.^137^ In addition, the crystal lattice of CaF_2_ exhibits low-phonon energy properties, enabling the doping of multiple lanthanide ions for anticancer investigations.^138,139^

Accompany with the therapeutic effects, calcium-based nanomaterials can also enhance multi-modal guided diagnosis. With the accumulation of calcium, the tumour tissue will be calcified, leading to a high-attenuated CT signal. The calcium nanoparticle-related calcification has shown enhanced CT imaging efficacy for a variety of mouse tumour models.^122,140–142^ Indeed, tumour calcification is regarded as a sign to predict the prognosis in clinic. For example, metastatic colorectal cancer patients with tumour calcification is associated with better treatment outcomes.^143^ In bone cancer, calcified tissue with a smooth and contiguous zone at the periphery of the tumour is indicative of good response to chemotherapies.^144^ The calcification also restricts the suspected zone of tumour boundaries, helping to verify the tumour-free tissue to preserve. In patients without calcification, dissection of some susceptible margin areas is required to ensure the completion tumour removement.^144^ However, calcification is not applicable in the post treatment prediction of bone cancer, as the lesion site will be removed directly by surgery. According to these notions, calcium nanoparticles with specific targeting calcium delivery may benefit bone cancer patients to optimise the operation plans through pre-treatment. Apart from CT imaging, some calcium nanomaterials are candidate contrast agents in other aspects. CaCO_3_ can be applied for enhancing the ultrasound imaging signals due to its ability of CO_2_ bubble generation under acidic pH conditions. Without coating, the dissolution of nanoscale CaCO_3_ can happen in mild acidity conditions (pH 6.8-7.2), enabling the TME-responsive bubble production to enhance echogenic signals.^145,146^ Core-shell structure nanoparticles with a CaCO_3_ core and polymer shells are able to protect against extracellular dissolution and achieve a intracellular bubble generation at an endo-lysosomal pH range (6.5-5.0).^147,148^

**Fig. 6:**
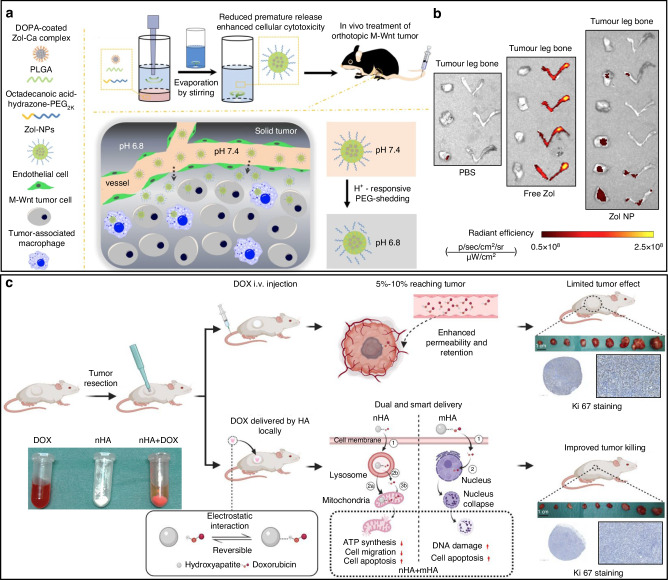
Calcium-based nanocarriers for bone-related anticancer agent delivery. **a**, **b** The Zoledronate, as a bisphosphate drug, formed calcium-based nanomaterials as Zol-NP to treat bone cancer. The Zol-NP showed retained Zol release and increased cytotoxicity to tumour cells and macrophages. Compared with free Zol, the Zol-NP showed significantly decreased Zol accumulation in bones, increased Zol accumulation in tumour and tumour inhibition.^151^ (**c**) The nHA (nano size) and mHA (micro size) were formed by loading doxorubicin (DOX) to hydroxyapatite to treat tumour. The locally delivery of DOX by HA showed improved anticancer efficacy comparing with intravenous injection of DOX. The released DOX from nHA through endocytosis by lysosome and unreleased DOX were delivered to mitochondria and resulted in insufficient ATP synthesis, less cell migration and more apoptosis. The released DOX from mHA extracellularly was up-taken in nucleus and caused more DNA damage and cell apoptosis.^154^ (Reprinted with copyright permissions)

Apart from the above intrinsic properties, calcium-based nanomaterials are widely applied as a delivery platform for therapeutic agents. They are featured with the high affinity to a wide range of bioactive molecules, including the widely used anti-osteoporosis drug BPs.^149,150^ Calcium ions are easily to bind with the phosphonate groups on such drugs. As shown in Fig. 6a, zoledronate, a third generation bisphosphonate, was engaged with calcium to fabricate a nanoparticle formulation denoted as Zol-NPs.^151^ We also reported a similar calcium-BP based nanoparticles named Ca-RIS NPs, showing an excellent penetration ability to bone tissues.^152^ With TME responsive abilities, the Zol-NPs showed significant accumulation to the orthotopically transplanted mammary tumour in the mice model, whereas the free zoledronate were more likely for bone accumulation (Fig. 6b). Taking advantage of the calcium-nanomaterials, the platform shed lights on the treatment of extraskeletal tumour with BPs. The payload of anticancer agents augments the anticancer ability of calcium nanomaterials. This payload can be achieved via physical absorption and chemical crosslink/co-precipitation.^153^ As an example shown in Fig. 6c, a paradigm chemotherapy agent doxorubicin (DOX) was loaded to nanoscale and macroscale hydroxyapatite to form n-HA and m-HA.^154^ These particles ameliorated the drug accumulation in mice with subcutaneous osteosarcoma tumour and induced effective tumour killing via mitochondrial dysregulation and nucleus collapse.

## Bisphosphonate based nanodrugs and bone targeted nano-delivery

### BP family drugs for osteoporosis and bone cancer treatment

**Fig. 7:**
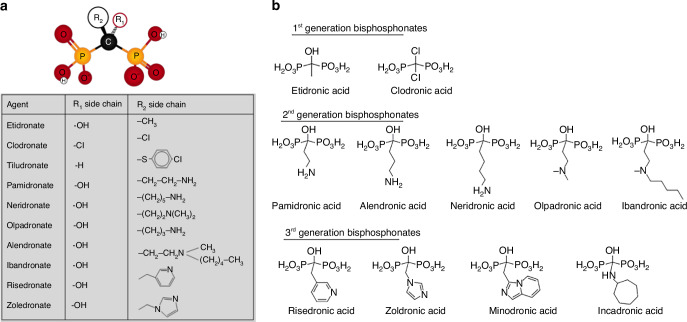
Chemical structure overview of BP family members. **a** BP chemical structure and groups of some common family members. **b** The representative members in different generations of bisphosphonates in acid forms

BPs are the most commonly prescribed treatment for osteoporosis^155^ and have been shown to be safe for long-term use,^156^ unlike hormone replacement therapy or RANKL pathway inhibition. BPs are a small molecule with a backbone of phosphorus and carbon atoms (Fig. 7a). They were first identified as regulators of calcification and bone resorption and reported as an osteoporosis therapeutic in rat models in 1971.^156,157^ BPs are also a commonly used drug in clinics to treat bone metastatic cancers and in these settings, high dose and frequent use are needed resulting in side effects.^158,159^

BPs are categorised into three generations based on their chemical structure. The first-generation BPs contain no nitrogen atoms (Fig. 7b), such as the first reported member - etidronate and clodronate. These early-generation BPs can metabolically transform into non-hydrolysable ATP analogues, thereby disrupting the ATP-dependent processes of the target cells. The second-generation BPs contain a nitrogen moiety (Fig. 7b) that enables them to target the farnesyl pyrophosphate synthase enzyme and disrupt the mevalonate pathway. This mechanism offers enhanced treatment efficacy compared to first-generation BPs. The 2nd-generation BPs include pamidronate, alendronate, neridronate, olpadronate and ibandronate. The third generation of BPs (Fig. 7b) were synthesised with a nitrogen-containing heteroaromatic group to enhance the enzyme-inhibition property, represented by risedronate and zoledronate.^156,160,161^

BPs can induce osteoclast apoptosis, thereby inhibiting bone resorption.^162^ BP treatment significantly increases bone mineral density in patients with osteoporosis by promoting bone healing, suppressing bone turnover and enhancing bone remodelling.^163–165^ Among the BP family drugs, oral administration of Alendronate (ALN) and IV administration of Zoledronate (ZOL) are widely used for osteoporosis patients, while ZOL therapy showed a superior effect to increase bone mineral density compared to ALN.^166^

Primary bone tumours arise from the uncontrolled proliferation of bone tumour cells, including bone giant cell tumours, osteosarcoma, multiple myeloma and Ewing’s sarcoma.^167^ Furthermore, bone is a common cancer metastasis site, with up to 100% of prostate and 70% of breast cancer patients developing bone metastases.^168^ Therefore, bone cancer treatments are highly sought-after to prolong survival and improve the quality of life, while BPs have demonstrated great success as adjuvant therapies for bone cancers and reducing pain.^162,169^ BPs can directly induce tumour cell death in multiple cancer types in vitro and in vivo^170,171^ via multiple mechanisms, including tumour apoptosis, reducing the number of tumour-associated macrophages, activating γδ T cells, inhibiting HER1/2 signalling and anti-angiogenic properties.^172–175^ BPs are used to treat primary bone tumours such as OS,^176^ which can improve cancer prognosis in combination with chemotherapy.^177^ BP treatment has also shown similar improvements in primary chondrosarcoma, where it reduced osteoclast-mediated bone destruction and decreased tumour size.^178^ In mouse models, BP (ZOL) inhibited primary Ewing’s Sarcoma tumour development in bone^179^ and reduced the ability of cancer cells to spread to the lungs.^180^

In addition to bone cancer treatment, BPs can delay bone metastasis of cancer and reduce tumour growth in some metastatic patients. For example, zoledronate prevents skeletal-related events (SREs) in non-small cell lung cancer by delaying their onset and reducing their number in the clinic.^181^ The BP family drugs also prevent the spread of prostate cancer by reducing levels of SREs and slowing disease progression.^182–184^ In the meantime, BP (ZOL) treatment relieved pain and reduced bone lesions for patients with unresectable benign tumours.^185^ BP treatment also prevents and palliates pain during the onset of SREs.^181^

Administering BP can cause several adverse effects,^159^ including (a) short-term effects of musculoskeletal pain, upper gastrointestinal track issue acute phase reaction, hypercalcemia and ocular inflammation and (b) long-term effects of atrial fibrillation, jaw osteonecrosis, subtrochanteric femoral fractures and suppressed bone turnover.^186,187^ In summary, the BP family drugs are a proven treatment for primary bone cancer and bone metastasis cases. However, they have not shown a significant treatment effect for breast cancer,^188^ indicating that their anti-cancer properties may be limited to some bone cancers. The reason for this is that BPs are small molecule drug with a half-life time in blood of only 2 h.^189^ Nanotechnology presents a possible solution to increase their circulation half-life and thus increase drug efficacy.

### BP based nanodrug and nano-delivery

Anticancer drugs generally accumulate poorly at bone tumour sites, causing systemic adverse effects when distributed non-specifically. Drugs further need to penetrate the dense extracellular matrix of bone tissues hindering their ability to interact directly with bone cells.^190,191^ Developing targeted delivery systems of anticancer drugs for bone tumours is essential. In addition to the osteoporosis and bone cancer therapy functions, the BP family has great bone tissue targeting properties. Bone tissue contains 50%–70% of metal minerals, primarily calcium phosphate.^192^ Due to the bidentate binding between the two phosphonate groups on BPs and the calcium ions, the BP molecules have a high affinity to bone tissues.^193^ Among BP family members, ZOL has the highest binding affinity for calcium phosphate, followed by ALN, ibandronate, risedronate, etidronate and clodronate.^194^ Thereby, ZOL and ALN are the most popular target moieties in the design of bone-targeted therapeutics, including BP-drug conjugates,^195^ polymeric micelles,^196^ inorganic nanoparticles,^197^ and engineered cells.^198^ Overall, the ZOL modification and ALN conjugation increased the accumulation of nanoparticles in bone tissues by up to 50-fold and 8-fold, respectively.^199,200^ Additionally, other BPs, such as risedronate (RIS),^152^ pamidronate,^201^ and neridronate,^202^ have been investigated for bone-targeted delivery. Recently, the BPs-modified lipid nanoparticles (LNPs) were reported for bone-targeted delivery of mRNA. Following in vitro screening of BP-LNPs formulations, Mitchell, et at.^203^ identified a lead BP-LNP formulation with enhanced (2.5-fold) mRNA expression in the bone in vivo compared to 490-C14 LNPs in the absence of BPs. Our group reported the synthesis of Calcium-Risedronate NPs (Ca-RISNPs) as a new nano-drug and drug delivery system for bone diseases and cancers.^152^ These Ca-RISNPs exhibit remarkable tissue penetration and uniform distribution in human bone and cartilage tissues, leading to superior drug and drug delivery to bone cells. Notably, these risedronate-containing NPs also demonstrate high specificity in eliminating tumour-associated macrophages (TAMs) and effectively inhibit TAM-induced tumour cell migration. This technique can be further explored for other BPs as nano-drugs and delivery systems.

In addition to BP modification, other bone-targeting moieties have also been reported, such as aspartic acid (Asp)-rich peptides and synthetic phosphorous-containing polymers. Firstly, the Asp-rich proteins (e.g. osteocalcin and osteopontin) showed high affinity to bone tissues, which inspired the studies of applying Asp-rich peptides as bone-seeking agents.^204^ After Asp-rich peptide modification, the bone accumulation of NPs could be increased by more than 10-fold.^205,206^ In general, the Asp-rich peptides performed weaker as ligands for bone-targeted delivery than BPs, though they have the advantage of convenient synthesis and easy for chemical modification.^207^

Secondly, various synthetic phosphorus-containing molecules and polymers have been developed for bone-targeted therapeutics utilising the similar calcium-binding mechanisms of BPs. For instance, by attaching bisphosphonate groups, the polyethene glycol (PEG) demonstrated bone-targeting properties,^208^ which has the potential for treating breast cancer bone metastases.^209^ Pyrophosphorylation is another strategy for bone-targeted drug delivery since Liu et al.^210^ demonstrated the therapeutic potential of pyrophosphorylated cholesterol-modified liposomes in the treatment of delayed bone fracture union. Though these phosphorus-containing molecules demonstrated targeting abilities, they were less effective than BPs. The bisphosphonate-modified PEG and pyrophosphorylated liposome exhibited less than a 5-fold increase in bone distribution compared to their non-modified groups.^209,210^

### Bifunctions of BP based nano carriers

In addition to their bone-targeted delivery property, the BPs conjugated on nanoparticles retained their inherent anti-osteoporosis and anti-cancer bioactivities. For example, ALN-modified nanoparticles inhibited bone resorption,^211^ while ZOL-engineered nanoparticles showed anti-proliferative effects on cancer cells.^212^ Therefore, BPs could be incorporated into nanomaterials simultaneously serving as targeting ligands and drugs. This dual functionality would represent a strategic approach to maximise the efficacy of BPs.

Moreover, the alendronate modification enhanced the bone accumulation of iron oxide nanoparticles, which scavenged the ROS in the target site. This facilitated the modulation of local immunity in bone tissues, creating a favourable environment for positive regulation of bone metabolism as described above. Combined with BPs, this nano-drug promoted osteogenic differentiation while inhibiting osteoclast differentiation.^213^ Another successful design is the bisphosphonate-based coordination polymers (BPCPs). The bioactive metals (Ca^2+^, Zn^2+^ and Mg^2+^)-absorbed BPCPs have channels to encapsulate an antineoplastic drug (letrozole, LET), which allows the combination of LET with BPs to treat breast cancer-induced osteolytic metastases (OM). The Ca^2+^ doped BPCPs (Ca@BPCPs) demonstrated the potential as drug-delivery systems to treat OM or other bone-related diseases because of their higher affinity to bone.^214^

In addition, we have recently reported that BP (ALN) can be used to stabilise CaP nanoparticles (named BCP NPs) and these NPs can effectively deliver plasmid DNA expressing ovalbumin (OVA) to generate strong antibody responses to OVA and immunity against OVA-expressing tumours.^215,216^ Though the distribution of these NPs in bone was not described in the studies, they are assumed to have the ability to deliver gene therapy (DNA or RNA) to bone and generating robust immunity. In summary, these studies showcased the promise of utilising BPs in nanoparticles as both targeting and treatment components. BP-containing NPs are a promising new approach to the treatment of bone diseases and cancers.

## Other bone targeted nano-delivery systems

Another big category of bone-targeting delivery systems is directly targeting the biomarkers of bone diseases including bone metastatic cancers and the delivery of specific therapeutics to bone diseases.

### Targeting steroid hormones

Steroid hormones are closely related to bone development and maintenance and the metastasis of cancers related to reproductive systems like prostate and breast. For instance androgen is essential for bone development and the maintenance, conditions with androgen deficiency, such as male hypogonadism androgen-insensitive syndromes and prostate cancer with androgen deprivation therapy are strongly associated with bone loss and increased fracture risk.^217^ It is also evident that androgen therapy benefits the skeletal more than male sexual hormones.^217^ A 17-β estradiol (E2) molecule was reported to co-load in a PLGA nanoparticles-based drug delivery with iron oxide (Fe3O4) and ALN modifications to achieve bone targeting and realise a magnetically remote-controllable drug release. The NPs were effective in enriching drugs in bone tissue and when exposed to an external magnetic field ameliorated OVX-induced bone loss improved bone strength, increased PINP and OC and downregulated CTX and TRAP-5b with fewer side effects on non-skeletal tissues.^218^ Previously, when the E2-loaded PLGA nanoparticles were administrated once a week, bone mineral density was significantly higher than that of the non-treated group at 60 days after the start of treatment. Also, in the group administered this NP twice a week, the bone mineral density increased significantly at 45 days after the start of treatment.^219,220^ Hormone replacement therapy (HRT) with E2-loaded PEGlyated up-conversion NPs (E2-UCNP@pPEG) were reported to retain E2 bioactivity and improve delivery efficiency in bone over a relatively long time-period.^221^ These data suggest that these delivery systems for hormone-like drugs can be considered as a potential therapeutic agent for osteoporosis.

### Targeting bone disease specific biomarkers

Some studies reported the direct targeting of biomarkers of bone diseases. A typical example for osteosarcoma is targeting cancer cell surface markers of B7-H3, GD2 and ER2 with antibody-drug conjugates (ADC).^222^ PLK1 gene was also reported as an excellent target to effectively inhibit osteosarcoma U2OS cell growth after the gene silence with a siRNA delivered by timely polymer NPs with a cytotoxicity as high as 90%.^223,224^ For OA, ERK was also shown to be a critical factor linking to its development^225^ and gene silence of ERK with the same polymeric NPs loaded siRNA could slow down the progress of OA.^223^ In addition, TGF-β was reported to be an important factor for OA development and a promising therapeutic target for the disease,^226^ but no nano-delivery data have been reported yet.

Some specific miRNA including four novel ones that play important roles in subchondral bone pathogenesis of OA have been identified by us^227^ and some were studied by Gao Y et al. ^228^ indicating that certain miRNAs are closely associated with bone disease development and progress thus can be targeted for treatment. For example, miR-181a is an oncomiR that is overexpressed in high-grade chondrosarcoma that promotes tumour progression. Regulator of G-protein signalling 16 (RGS16) is a target of miR-181a. Inhibition of RGS16 expression by miR-181a enhances CXC chemokine receptor 4 (CXCR4) signalling, which in turn increases MMP1 and VEGF expression, angiogenesis and metastasis.^228^ Sun et al. ^229^ reported the systemic treatment with anti-miRNA oligonucleotides (AMO) directed against miR-181a utilising a nanopiece delivery platform. This platform was combined with a molecular beacon or anti-miR-181a oligonucleotides and were shown to transfect chondrosarcoma cells in vitro and in vivo intra-tumoral injection and systemic delivery had similar effects on miR-181a expression in nude mice bearing chondrosarcoma xenografts. Systemic delivery of NP carrying anti-miR-181a also restored RGS16 expression, decreased expression of VEGF and MMP1, MMP activity and tumour volume by 32% at day 38 and prolonged survival from 23% to 45%.

For RA, dexamethasone (DEX) is a commonly used agent for its therapy on inflammation manage, but the traditional administering DEX is hampered by low efficiency and adverse effects. Zhao et al. ^230^ developed transdermal formation dextran sulphate (DS) modified DEX-loaded flexible liposome hydrogel (DS-FLs/DEX), validated their transdermal efficiency, evaluated its ability to target activated macrophages and its anti-inflammatory effect. The DS-FLs/DEX exhibited excellent biocompatibility, sustainable drug release and high uptake by lipopolysaccharide (LPS)-activated macrophages. Furthermore, the DS-FLs/DEX showed desired skin permeation as compared with regular liposome hydrogel (DS-RLs/DEX) due to its good deformability. In vivo, when used the AIA rats as RA model, the DS-FLs/DEX hydrogel can effectively penetrate and accumulate in inflamed joints, significantly improve joint swelling in RA rats and reduce the destructive effect of RA on bone. Moreover, the expression of inflammatory cytokines in joints was inhibited and the system toxicity did not activate under DS-FLs/DEX treatment. Because the diversity and complex of the bone diseases, drug delivery can be tail designed to suit the individual conditions. A review article summarised the advanced applications of triggerable nanomaterials dependent on various internal stimuli (including reduction-oxidation (redox), pH and enzymes) and external stimuli (including temperature, ultrasound, magnetic, photo, voltage and mechanical friction). The review also explores the progress and challenges with the use of stimuli-responsive nanomaterials to manage inflammatory arthritis based on pathological changes, including cartilage degeneration, synovitis and subchondral bone destruction. Exposure to appropriate stimuli induced by such histopathological alterations can trigger the release of therapeutic medications, imperative in the joint-targeted treatment of inflammatory arthritis.^231^ This is the future direction of bone targeted nano-delivery.

### Targeting specific markers of bone metastatic cancers

Some cancers are specifically metastatic to bone, including prostate, breast and lung cancers. Therapies targeting specific marker of these cancers are specific to bone metastasis. However, so far there are not many studies in this area, the common nano-delivery system from available studies still replies on NPs grafted with calcium and BP as targeting motifs loaded with anti-cancer drugs. In addition, studies also focused on targeting the bone microenvironment, such as reshaping the macrophage phenotypes.^232^

#### Prostate cancer

A PTX-PEG-ALN nanoparticle was designed and shown to have a great binding affinity to the bone mineral hydroxyapatite in vitro and the nano-delivery exhibited an improved pharmacokinetic profile compared with the free drugs owed to the marked increase in their half-life.^233^ A similar NPs were developed using poly (D,L-lactic-co-glycolic acid) and cabazitaxel (CBZ) as the core with amino-bisphosphonate surface conjugation to test in vitro in prostate cancer cell lines and in vivo in an intraosseous model of metastatic prostate cancer. This bone targeted CBZ nanocarrier system showed significant reduction in tumour burden, while at the same time maintaining bone structure integrity and reducing pain in the mouse tumour limb.^234^

Castration-resistant prostate cancer (CRPC) accounts for most prostate cancer deaths and patients with CRPC are readily developing chemo drug resistance. Zhang et al. ^235^ reported a codelivery of a chemotherapeutic agent and a siRNA as advanced strategy to treat drug-resistant prostate cancer. RGD-PEG-DSPE/CaP nanoparticles were further modified to obtain the LCP-RGD nanoparticles that contain a calcium phosphate (CaP) core, dioleoyl phosphatidic acid (DOPA) and RGD modified poly(ethylene glycol)-conjugated distearoyl phosphatidylethanolamine (RGD-PEG-DSPE). This drug delivery system was used for codelivery of GRP78 siRNA and docetaxel (DTX) for the treatment of the CRPC PC-3 cells. The CaP core can compress the negatively charged siRNA, while the DOPA and RGD-PEG-DSPE component can effectively carry DTX. The arginine-glycine-aspartic acid (RGD) can target the prostate cancer cells. The study showed that the codelivery of DTX and GRP78 siRNA had an in vitro and in vivo combinational anti-prostate cancer effect and could sensitise the cell-killing effect of DTX; which is especially suitable for drug-resistant CRPC treatment. Similarly, Haong et al. ^236^ report the development of a polymer-based delivery system for CBZ to improve its safety and efficacy against DTX-resistant mCRPC. CBZ was conjugated to a carboxymethylcellulose-based polymer (Cellax-CBZ), which self-assembled into ∼100 nm NPs in saline and exhibited sustained drug release in serum at 10%. Cellax-CBZ delivered 157-fold higher CBZ to PC3-RES prostate tumour in mice and could be safely administered at a 25-fold higher dose compared to free CBZ, resulting in superior tumour inhibition in multiple mouse models of DTX-resistant CRPC. In a metastatic bone model of CRPC, Cellax-CBZ significantly improves overall survival with a 70% long-term survival rate to day 120, while mice treated with free CBZ had a median survival of 40 days. Cellax-CBZ induced mild and reversible neutropenia in mice but no other tissue damages.^236,237^

Besides, Barbanente et al. ^238^ reported selenite-doped hydroxyapatite nanoparticles loaded with a hydroxyapatite-binding anti-tumour platinum complex (PtPP-HASe) selectively reduce proliferation of PC3 cancer cells without reducing proliferation of bone marrow stem cells. These PtPP-HASe particles were nanocrystalline with selenium (Se) and platinum (Pt) contents ranging between 0–10 and (1.5–3.0) wt%, respectively. At a Pt/Se ratio of 8, released Pt and Se species selectively reduced cell number of PC3 and breast cancer cells MDA-MB-231 by a factor of >10 with limited effects on co-cultured human bone marrow stem cells (hBMSc), demonstrating high anti-cancer selectivity and offer ample opportunities for the design of novel biomaterials with potent and selective chemotherapeutic efficacy against prostate cancer.^238^ Evans et al. ^239^ reported anisamide-targeted NPs that targets the sigma receptor positive cells. When cells were grown on 3D scaffolds, recapitulating bone metastasis, targeted formulations showed significantly higher levels of PLK1 mRNA knockdown (46% for PC3 and 37% for DU145, *P* < 0.05). This is the first time that a targeted cyclodextrin has been used to transfect prostate cancer cells in a 3D model of bone metastasis.^239^

A disulphide cross-linked arginine-aspartic acid peptide modified by HAIYPRH (T7) was reported to have specific affinity to prostate cancer cells. The peptide T7-modified polypeptide NPs for delivery DNA (CRD-PEG-T7/pPMEPA1) was prepared and tested for their cell uptake.^240^ The cellular uptake of LNCaP cells was decreased after treating with excessive free T7, endocytosis inhibitors and lower incubation temperature. Besides, CRD-PEG-T7/pPMEPA1 could inhibit the LNCaP cells chemotaxis and tumour growth. The survival duration of the PCa tumour-bearing mice treating with CRD-PEG-T7/pPMEPA1 was significantly prolonged.^241^

Katsumi et al. ^242^ reviewed the approaches that using antibodies to prostate-specific membrane antigen (PSMA) in active targeting bone metastatic prostate cancer. Some conjugates using antibodies to PSMA were developed and used in clinical trials. In addition, recent challenges in the development of bone-targeted delivery systems and strategies for the treatment of bone metastasis have been summarised in this review.^242^ One of the studies reported that RNA aptamer (APT) A10-3.2 has been used as a ligand to target PCa cells that express PSMA.^243^ APT was investigated as a PSMA-targeting ligand in the design of an ATE-based microRNA (miRNA; miR-15a and miR-16-1) vector to PCa bone metastasis. To observe the targeted delivery and transfection efficiency of ATE-APT in PSMA-overexpressing cells, luciferase activity and biodistribution of nanoparticles in Balb/c mice was analysed. The anticancer effect of nanoparticles in vivo was investigated using the survival times of human PCa bone metastasis mice model. Luciferase assays of pGL-3 expression against PC3 (PSMA^-^) and LNCaP (PSMA^+^) cells showed that the transfection efficiency of the synthesised DNA/ATE-APT complex was higher than that of the DNA/ATE complex. The anticancer efficacy of miRNA/ATE-APT was superior to those of other treatments in vivo.^243^ This PSMA-targeted system may prove useful in widening the therapeutic window and allow for selective killing of PCa cells in bone metastasis.

Moreover, two review papers summarised about targeting tumour niche in bone to inhibit bone metastatic cancers.^244,245^ The bone microenvironment can inhibit the growth of disseminated tumour cells (DTC) by inducing dormancy of the DTC directly and later following formation of a micrometastatic tumour mass by inhibiting metastatic processes including angiogenesis, bone remodelling and immunosuppressive cell functions, highlighting some of the mechanisms mediating DTC dormancy and the complex relationships occurred between tumour cells and bone resident cells in the bone metastatic microenvironment. It is vital to understand the inter-cellular interactions that have important targets to be considered for the development of novel effective therapies for prevention or treatment of bone metastases.

#### Breast cancer

Studies using different NPs (silica NPs, polymer NPs and even silk fibres) to deliver DOX, CTP, or BP to treat bone metastatic breast cancer. Among them, BP and calcium are still usually used as the bone targeting agents to specifically deliver to bone. However, some other targets like migrating molecule CXCR4, integrin receptors, HER2 receptor, bone cells and even tumour niche are reported.

Li et al. ^246^ reported that the DTX based therapy increases the metastasis risk due to the upregulation of CXCR4 expression during the treatment. They conjugated CXCR4 antagonist peptide (CTCE) with DTX (termed CTCE-DTX) as an anti-metastasis agent to treat breast cancer. CTCE-DTX could self-assemble to NPs, targeting CXCR4-upregulated metastatic tumour cells and enhancing the DTX efficacy. Thus, the CTCE-DTX NPs achieved inhibition of both bone-specific metastasis and lung metastasis of triple-negative breast cancer.

Integrin receptors play an important role in cancer development and communication with ECM. Some integrin is more specifically express on breast cancer that led to bone metastasis. Ross et al. ^247^ provided evidence that integrin β3 (β3) is selectively induced on breast cancer cells in bone by the local bone microenvironment. In a preclinical model of breast cancer, β3 was strongly expressed on bone metastatic cancer cells but not primary mammary tumours or visceral metastases. In tumour tissue from breast cancer patients, β3 was significantly elevated on bone metastases relative to primary tumours from the same patients (*n* = 42). Using a micelle-based nanoparticle therapy that recognises integrin αvβ3 (αvβ3-MPs of ~12.5 nm), they demonstrated specific localisation to breast cancer bone metastases in mice. Using this system for targeted delivery of the DTX, they showed that bone tumour burden could be reduced significantly with less bone destruction and less hepatotoxicity compared to equal doses of free DTX. Furthermore, mice treated with αvβ3-MP-docetaxel exhibited a significant decrease in bone-residing tumour cell proliferation compared to free docetaxel.^247^ A similar study using mTORC1 as a candidate for therapeutic targeting of this β3-mediated, chemotherapy-induced metabolic response. mTORC1 inhibition in combination with docetaxel synergistically attenuated murine bone metastases. Further, micelle nanoparticle delivery of mTORC1 inhibitor to cells expressing activated αvβ3 integrins enhanced DTX efficacy in bone metastases.^248^ These data indicate that αvβ3 integrin can be explored as a selective marker for breast cancer cells within the bone. In addition, Katsumi et al. ^242^ reviewed the approaches that antibodies to human epidermal growth factor receptor 2 (HER2) are used in active targeting bone metastatic breast cancer. Some conjugates using antibodies to HER2 were developed and used in clinical trials.^242^

Like prostate cancers, targeting bone cells or metastatic niche were explored, which aimed drug delivery into osteogenic niches to inhibit DTC colonisation can prevent bone metastasis from entering its late stage and therefore cure bone metastasis. Liu et al. ^249^ constructed a 50% DSS6 peptide conjugated nanoparticle to target the osteogenic niche. The osteogenic niche was always located at the endosteum with immature hydroxyapatite. Arsenic-manganese nanocrystals were loaded in osteogenic niche-targeted PEG-PLGA nanoparticles with an acidic environment-triggered arsenic release. Arsenic formulations greatly reduced 4T1 cell adhesion to mesenchymal stem cells (MSCs)/preosteoblasts (pre-OBs) and osteogenic differentiation of osteoblastic cells. Arsenic formulations also prevented tumour cell colonisation and dormancy via altering the direct interaction between 4T1 cells and MSCs/pre-OBs. The chemotactic migration of 4T1 cells toward osteogenic cells was blocked by arsenic in mimic 3D osteogenic niche. Systemic administration of osteogenic niche-targeted arsenic NPs significantly extended the survival of mice with 4T1 syngeneic bone metastasis.^249^ These findings provide an effective approach for osteogenic niche-specific drug delivery and suggest that bone metastasis can be effectively inhibited by blockage of tumour cell colonisation in the bone microenvironment.

#### Lung cancer

Only a few studies reported the delivery of TDX for treating bone metastatic lung cancer. A study showed the pH-sensitive nanoparticle composed of ALN and poly(amidoamine) (PAMAM) that was loaded with docetaxel (DTX@ALN-PAMAM).^250^ The in vitro results showed DTX@ALN-PAMAM significantly enhanced the anticancer activity of DTX and inhibited the formation of osteoclasts. DTX@ALN-PAMAM concentrated at bone metastasis site in mice, which resulted in the suppression of bone resorption, pain response and growth of bone metastases. Similarly, Bai et al. ^251^ reported a DTX-loaded nanoparticle, DTX@AHP (ALN-hyaluronic acid-PAMAM), which could target dually at osteoclasts and bone metastatic tumour cells. The in vitro drug release from DTX@AHP exhibited pH and redox responsive characteristics. DTX@AHP displayed high binding affinity with bone matrix. In addition, DTX@AHP significantly inhibited the differentiation of RAW264.7 into osteoclast and effectively inhibited the proliferation of osteoclasts and tumour cells in in-vitro 3D bone metastases model of lung cancer. DTX@AHP could accumulate in bone metastases sites in vivo. Consequently, DTX@AHP not only markedly inhibited the growth of bone metastases of lung cancer but also reduced osteolysis in tumour-bearing mice.^251^

### Bone exosome delivery systems

Exosomes are extracellular nanovesicles secreted by many types of cells, they are used to treat diseases and widely used as drug delivery systems. Bone-derived exosomes are naturally existing nano-sized extracellular vesicles secreted by various bone cells, such as bone marrow stromal cells, osteoclasts, osteoblasts and osteocytes, containing multifarious proteins, lipids and nucleic acids. Accumulating evidence indicates that bone-derived exosomes are involved in the regulation of skeletal metabolism and extraosseous diseases through modulating intercellular communication and the transfer of materials. Following the development of research, exosomes can be considered as a potential candidate as a drug delivery carrier with its ability to transport molecules into targeted cells with high stability, safety and efficiency in bone.^252^ Apart from the various advantages, exosomes are standing out of the crowd for their ability to conduct cellular communication. The internal cargo of the exosomes is leading to its potential use in therapeutics. Exosomes are being unravelled in terms of the mechanism as well as application in targeting various diseases and tissues. The potential of the exosomes towards their contribution to the drug delivery scenario in the bone microenvironment is significant with a comparison of the pros and cons of exosomes with other prevalent drug delivery systems.^253^ The constructive strategies of modified exosomes to improve bone-targeting and their therapeutic effects for bone-related diseases are reviewed with a summary of the developments and challenges in bone-targeted exosomes.^254^

### Other reported systems

Some other ligands with bone-targeting properties have been explored. For example, the phenol- and hydroxyl-containing tetracycline exhibits a strong affinity for bone calcium phosphate. Tetracycline-conjugated nanoparticles have demonstrated an enhanced bone distribution.^255^ However, tetracycline’s side effects, including skeletal growth inhibition, limit its clinical application.^191^ Recently, bone-targeting research explored a peptide (TPLSYLKGLVTVG) with high affinity to a bone resorption surface protein (Tartrate-resistant acid phosphatase) has been identified as a promising bone-targeting ligand.^256^

## Perspective for bone targeted delivery and therapy

### Calcium and BP based nanodrugs and delivery systems

Most bone diseases company with bone loss thus calcium and BP based bone targeting delivery systems have advantages to not only provide targeting motifs but also strength the bone. Different calcium compounds offer unique features in calcium metabolism and osteogenesis or osteolysis thus NPs loaded with these compounds or calcium-based scaffolds warrant further and deep investigations to understand more on their regulatory roles in the microenvironment of bone to promote osteogenesis and the TME to inhibit tumour growth. Meanwhile, BP based nanodrugs and drug delivery systems donate a great promising for targeted treatments of bone diseases and offer more varieties for multifunctional NPs targeting bone. Among these, Ca-BP nanodrug and nano delivery for mRNA or DNA will attract more attention for bone gene therapy as using BP as targeting motifs has a possible disadvantage of BP loading capacity and release. Therefore, further exploration of Ca-BP nanodrugs or nano delivery systems for their preclinical/clinical benefits will boost the application of these nanomaterials. In addition, depending on the location of bone diseases, localised nano-delivery or systemic nano-delivery with disease responsive properties will be the future direction.

### Multi-functional NPs with dual targets

**Fig. 8:**
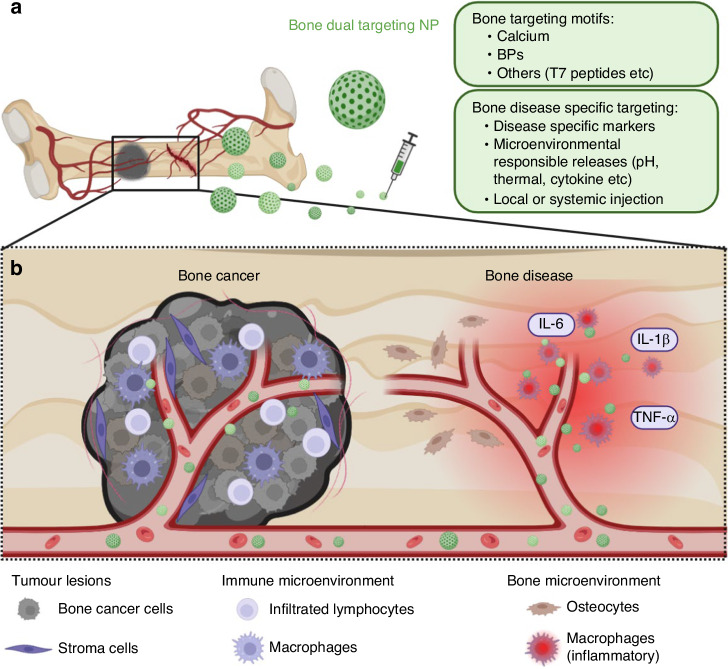
Perspective design of bone-targeting NPs for cancer and other bone diseases. **a** The bone dual targeting NPs with bone and disease specific targeting motifs can be administrated via local or systemic injections; **b** The dual targeted NPs can be directed to bone from blood vessel and further go to the precise tumour cells of the bone site to affect tumour cells and TME. If the NPs have a disease responsive property (like to heat or cytokines) they can also release the payload in situ. Figure created with BioRender.com

From above description, even with bone targeted nanodrug or nano-delivery, skeleton is widely distributed in our body, secondary target to deliver the drug specific to diseased cells or sites in bone is a necessity. To achieve this, the delivery system needs to have a long retention time in circulation and bone to give them more chance to disperse to specific disease sites. Therefore, the ideal delivery system will be able to go to the bone first (enough retention time in circulation) then are directed to disease cells or sites, the ratio and constructure of the two targeting motifs and release of them need more careful studies and to be optimised (Fig. 8).

The advantages of targeting both bone tissues and bone disease markers include the increased the specificity of delivery and reduced side effects to bone since most biomarkers for bone cancers or bone metastatic cancers are not unique to tumour cells. Moreover, solely bone targeted delivery can still randomly distribute to any bone tissue site in the body. Therefore, it is a strategical consideration about how to specifically target bone diseases with nano-delivery. So far there are only very few studies in this direction, but more research is expected in the future. To achieve this, the disease location is also an important factor to consider. If the disease location is localised or clearly known, such as the localised OA and RA and the site is accessible, the nano-drugs or nano-delivery systems can be bone targeted and locally administrated to avoid drug widespread. In addition, applying controlled release and using pH or themo-sensitive materials may be useful to release the drug in the disease sites more precisely. For bone diseases that the location is not clear or uncertain or usually spread to multiple sites like bone metastatic cancers, nano-drugs, or nano-delivery systems of multi-functional NPs with the dual targets to bone and cancer cell marker(s) becomes essential. In addition, the multi-functional NPs with bone targeting motifs and properties to responsive to bone disease microenvironment such as pH, temperature, cytokine secretions (Fig. 8) will be also expected.
